# Association between red blood cell transfusion and bronchopulmonary dysplasia: a systematic review and meta-analysis

**DOI:** 10.3389/fped.2023.1095889

**Published:** 2023-05-31

**Authors:** Li Tang, Ting Ting Zhu, Jing Zhao

**Affiliations:** ^1^ Department of Pediatrics Hematology and Oncology Nursing, West China Second University Hospital, Sichuan University, Chengdu, China; ^2^ Department of Pediatrics, West China Second University Hospital, Sichuan University, Chengdu, China; ^3^ Key Laboratory of Obstetric & Gynecologic and Pediatric Diseases and Birth Defects of Ministry of Education, Sichuan University, Chengdu, China

**Keywords:** bronchopulmonary dysplasia, transfusion, red blood cell, systematic review, meta-analysis

## Abstract

**Background:**

We aimed to determine the association between red blood cell transfusions (RBCT) and bronchopulmonary dysplasia (BPD) in neonates.

**Methods:**

A systematic review and meta-analysis were conducted using data obtained from literature search of PubMed, Embase, and Web of Science from their inception till May 1, 2022. Two reviewers independently selected potentially relevant studies, and after data extraction, they assessed the methodological quality of the included studies using the Newcastle–Ottawa scale. Data were pooled using random-effects models in Review Manager 5.3. Subgroup-analysis was performed based on the number of transfusions and adjusted results.

**Results:**

Of the 1,011 identified records, 21 total case-control, cross-sectional, and cohort studies were selected, which included a total of 6,567 healthy controls and 1,476 patients with BPD. The pooled unadjusted odds ratio ([OR], 4.01; 95% confidence interval [CI] 2.31–6.97) and adjusted OR (5.11; 95% CI 3.11–8.4) showed significant association between RBCT and BPD. A substantial heterogeneity was noted, which could be due to different variables controlled for in each study. The subgroup analysis showed that heterogeneity may be partially explained by the extent of transfusion.

**Conclusion:**

The association between BPD and RBCT remains unclear based on the current data due to the substantial heterogeneity among the results. Well-designed studies are still needed in the future.

## Introduction

Bronchopulmonary dysplasia (BPD) is a common complication in preterm infants. This complication can lead to asthma, wheezing, limitation of activity tolerance, and recurrent low respiratory tract infections (1, 2). Infants with lower birth weight (BW) and gestational age (GA) are at risk for developing BPD. In addition, maternal smoking, chorioamnionitis, male sex, oxygen therapy, duration of mechanical ventilation, and co-morbidities, including sepsis, necrotizing enterocolitis, and pulmonary hemorrhage have been found to be associated with BPD (3–5). Epidemiological studies have reported that approximately 80% of preterm infants born between 22 and 24 weeks of gestation will develop BPD to a certain degree (6). As a result of an increased survival rate of extremely preterm infants, the incidence of BPD has increased (7, 8); however, no specific or preventive treatment has developed.

Red blood cell transfusions (RBCT) are commonly used in neonatal intensive care units. A previous survey reported that the vast majority of extremely low BW infants and 58% of preterm infants (<32 weeks of GA) received RBCT in the neonatal period (9–11). However, the effects of RBCT on short and long-term outcomes remain unclear. Recent studies have reported an association between RBCT and intra-ventricular hemorrhage, retinopathy of prematurity, and necrotizing enterocolitis (12–14). Few observational studies have found that patients with BPD receive higher RBCT volume and the number of RBCT is associated with BPD development (15–17). Vecchio et al. instituted new guidelines for administering RBCT in their neonatal intensive care unit, and the transfusion rate was concordant with a lower incidence of BPD (18). However, other studies found no relationship between BPD and RBCT (19, 20). Therefore, we aimed to conduct a systematic review and meta-analysis of the available literature to investigate the association between RBCT and BPD.

## Methods

### Literature search

We performed this systematic review and meta-analysis in accordance with the Meta-analysis of Observational Studies in Epidemiology (MOOSE) and the Preferred Reporting Items for Systematic Reviews and Meta-Analysis (PRISMA) guidelines (21, 22). We searched the electronic databases PubMed, Embase, and Web of Science from their inception till May 1, 2022. The keywords “red blood cell,” OR “blood transfusion,” AND “BPD,” OR “bronchopulmonary dysplasia”, were employed as search term. Only articles published in English language were included. We also searched the reference lists of review articles for additional eligible studies. Detailed strategy for online database search is described in the supplemental files.

### Study selection

Studies were selected by screening titles, abstracts and full texts. Studies were included if they had a control group, and reported mean and standard deviation [(SD) or the data can be converted to mean and SD] of the number and total volume of RBCT between BPD patients and controls, as well as the odds ratio (OR) of RBCT for BPD. Case reports, letters to the editors, articles with incomplete data, and studies with less than 10 samples were excluded from analysis.

### Data extraction

One reviewer (J.Z.) extracted data from the selected studies into a predetermined form. The first author, published year, study design, country, group size, sample size, BPD diagnosis and transfusion data were recorded. Another reviewer (L.T.) checked the data to ensure the accuracy. Any disagreements were settled with a third reviewer (T.T.Z.).

### Quality assessment

Two authors (J.Z. and L.T.) independently evaluated the quality of each included study using the Newcastle–Ottawa scale (NOS) for case-control or cohort studies (23). This scale covers three parts of the study design: patient selection (4 points), comparability of the study groups (2 points), and exposure/outcome (3 points). A modified NOS (24) with a total of 10 points was used for cross-sectional studies. Studies with a score below six points were considered to be of low quality.

### Statistical analysis

We pooled the weighted mean difference and 95% confidence interval (CI) of total number of administered RBCT and total RBCT volume in patients with BPD and those without BPD, and the OR of RBCT for BPD, using a random effect model. A two-tailed *p* < 0.05 was considered statistically significant. Heterogeneity across studies was assessed using the chi-square test and *I*^2^. An *I*^2^ value > 75% indicated high heterogeneity. Publication bias was assessed visually based on the symmetry of funnel plots. Sensitivity analyses were performed by excluding one study at a time, to test the stability of the results. We calculated the mean and SD of the number or volume of RBCT scans using sample size, median and interquartile range (IQR) according to Hozo et al. (25), when needed. Data analyses were performed using Review Manager Software version 5.3.

## Results

A total of 1,011 articles were retrieved from the databases using systematic and manual searches. After removing the duplicates, and performing screening of abstracts and full-text articles, 21 studies (5, 15, 16, 19, 20, 26–41) were included. These studies included a total of 6,567 control infants and 1,476 patients with BPD, and were eligible for the meta-analysis. (Supplementary Material Figure S1). The characteristics of eligible studies are presented in Table 1.

**Table 1:** Characteristics of included studies.

Study ID	Published year	Country	Study design	Sample size (cases/control)	Included criteria	diagnosis of BPD	NOS score
Liao (26)	2021	China	Case-control	36/24	GA < 28w or BW < 1,000 g	Medical record	5
Duan (27)	2016	China	Cross-sectional	71/1,721	GA < 32 weeks	GA < 32w and need supplemental oxygen more than 28 days	5
Zhang (28)	2014	China	Cohort	137/94	GA ≤ 32w or BW ≤ 1,000 g	Oxygen dependency for at least 28 days	8
Demirel (19)	2009	Turkey	Case-control	56/50	BW < 1,500 g	Diagnosed according to the Bancalari criteria	6
Cai (29)	2021	China	Cross-sectional	63/388	GA < 32 weeks and BW < 1,500 g	Medical record	5
Sharma (30)	2019	USA	Cross-sectional	155/108	GA 23–27w	Oxygen dependency for at least 28 days	4
Lee (15)	2020	Korea	Cross-sectional	109/141	BW < 1,500 g	Persistent oxygen requirement at 36 weeks postmenstrual age	4
Ghirardello (31)	2016	USA	Cohort	269/372	BW < 1,500 g	According to the criteria proposed by Jobe and Bancalari at 36 weeks corrected age	7
Valieva (32)	2009	USA	Cross-sectional	47/5	GA ≤ 28w or BW 500–1,000 g	Requiring any type of oxygen support at both 28 days and at 36 weeks corrected GA	4
Patel (33)	2019	USA	Cohort	240/358	BW < 1,500 g	According to the consensus definition by the National Institutes of Health	7
Raffa (16)	2022	Arabia	Cohort	5/106	GA < 30 weeks and BW < 1,500 g	Medical record	6
Korhonen (34)	1998	Finland	Cohort	59/133	BW < 1,500 g	Requiring any type of oxygen support at both 28 days and characteristic radiographic changes in the lung fields	5
Jassem-Bobowicz (35)	2021	Poland	Cross-sectional	127/151	GA < 32 weeks	Oxygen dependency for at least 28 days	4
Tao (5)	2022	China	Cohort	102/523	Neonates with RDS	Requiring any type of oxygen support at both 28 days and at 36 weeks corrected GA	6
Soliman (36)	2016	Canada	Cohort	79/240	GA < 32 weeks and born to mothers with preeclampsia	Oxygen or any form of ventilation at 36 weeks’ postmenstrual age	6
Go (20)	2021	Japan	Cohort	85/91	GA < 30weeks	National Institutes of Health consensus definition for infants	7
Zhang (37)	2011	China	Cross-sectional	60/56	BW < 1,500 g	Oxygen for at least 28 days	4
Jeon (38)	2013	Korea	Cross-sectional	39/11	BW < 1,500 g	Oxygen dependency for the first 28 days of life	4
Gao (39)	2020	China	Cross-sectional	32/49	GA ≤ 28w or BW < 1,500 g	Oxygen for at least 28 days	4
Park (40)	2015	Korea	Cross-sectional	10/36	BW < 1,500 g	Required oxygen for the first 28 days at postmenstrualage 36 weeks or when infants born at ≥32 weeks and remained on oxygen for 56 days	4
Lardón-Fernández (41)	2017	Spain	Cohort	60/69	BW < 1,500 g	According to the consensus definition by the National Institutes of Health	7

W, week; g, gram; GA, gestational age; BW, birth weight; USA, United States of America.

All included participants had a GA of less than 32 weeks at birth or BW of less than 1,500 g. Studies included populations from different countries, with being China and the USA being the most common. Although all studies reported the association between BPD and RBCT, their objectives varied. In half of the included studies the primary objective was to investigate risk factors of BPD including RBCT. Two studies collected data based on medical records (16, 26), and others defined BPD as neonates requiring oxygen support at 28 days of life or at 36 weeks of corrected GA. Study design included cross-sectional (*n* = 10), cohort (*n* = 9), and case–control (*n* = 2).

The NOS scores of the included studies ranged from 4 to 8, and detailed information is provided in the supplemental files. In all included studies, exposed and non-exposed controls were drawn from the same community. However, common weaknesses were lack of adequate confounder adjustments such as neonatal sepsis, maternal diseases during pregnancy, and outcome assessment based on medical records without a description of blind.

**Figure 1:**
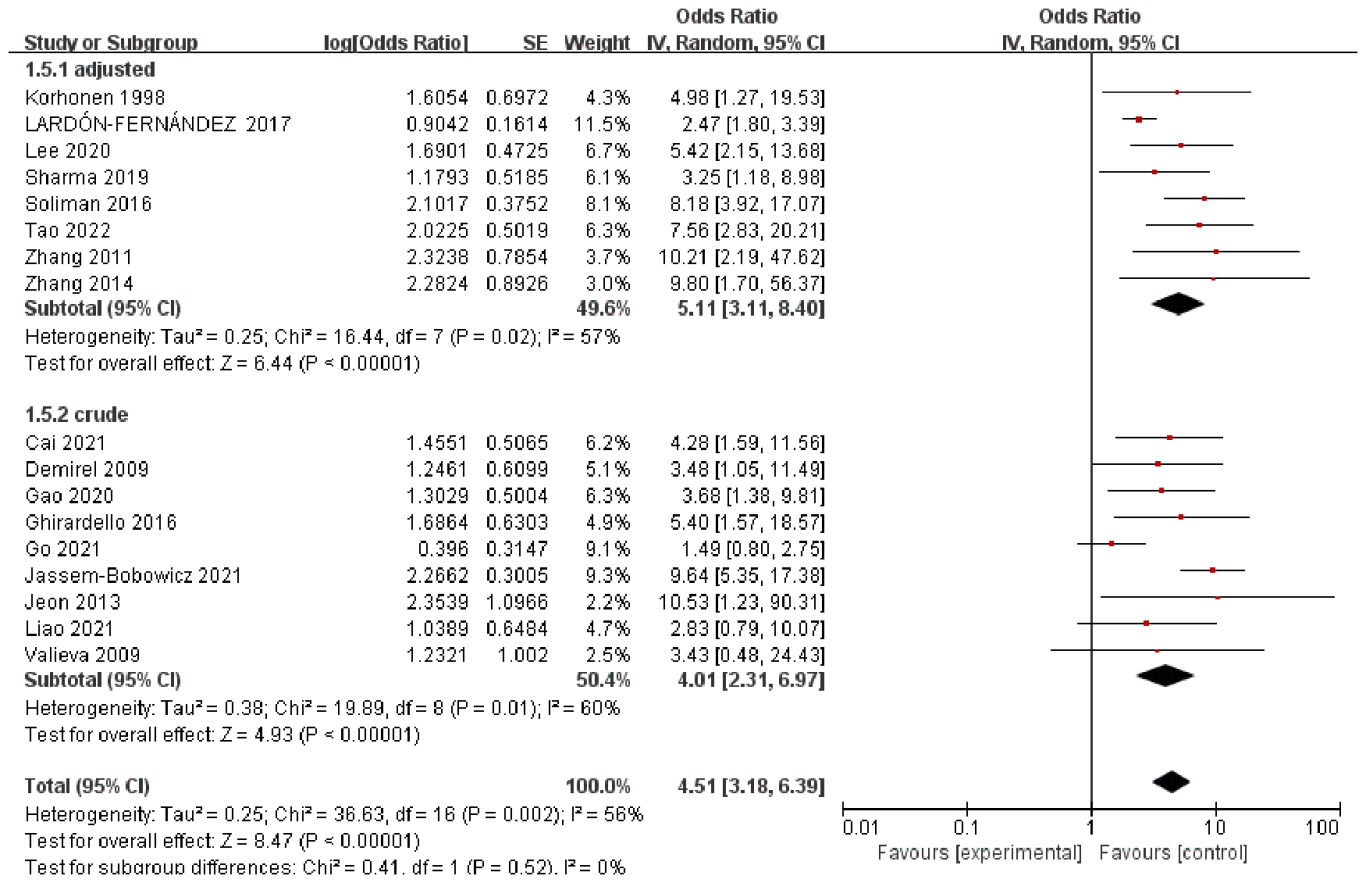
Forest plot for the association between RBCT and the development of BPD, stratified on crude or adjusted results.

**Figure 2:**
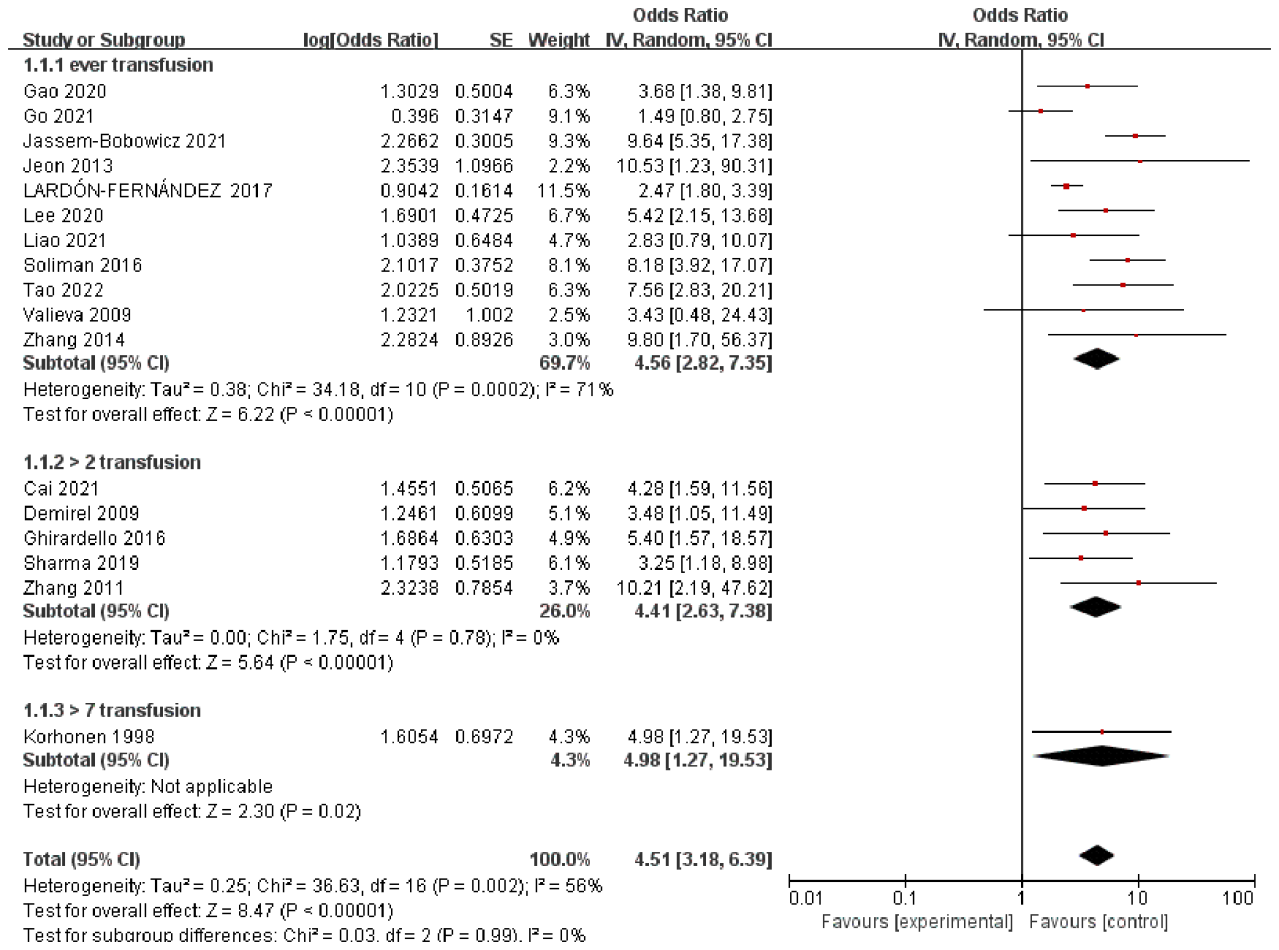
Forest plot for the association between RBCT and the development of BPD, stratified on number of transfusion.

The pooled ORs obtained using a random-effect model showed a significant association between RBCT and BPD in both crude analysis (OR 4.01, 95% CI: 2.31–6.97) and adjusted analysis (OR 5.11, 95% CI: 3.11–8.40). There was a substantial heterogeneity between studies (*I*^2 ^= 56%) (Figure 1). In addition, we conducted subgroup-analyses of the number of transfusions, and the association remained significant (Figure 2).

A pooled analysis of RBCT including 227 cases and 465 controls (Supplementary Material Figure S2) found that patients with BPD were more likely to have more RBCT than those without BPD (standard mean difference 2.62, 95% CI 0.99–4.24, *I*^2^ = 89%, *p* < 0.01).

Three studies including 255 cases and 500 controls were pooled for the total transfusion volumes. The standard mean difference of total RBCT volumes was 55.48 (95% CI: 3.82–107.4, *I*^2^ = 95%, *p *< 0.01) between patients with BPD and controls. (Supplementary Material Figure S2).

Except for the analysis of the total transfusion volume, there were no apparent changes in the pooled results when any single study was removed. Visual examination of the funnel plots of the studies included in this meta-analysis showed no apparent publication bias.

## Discussion

The aim of this systematic review was to evaluate the association between RBCT and BPD based on the available evidence to provide suggestions for future investigations. The meta-analysis of the unadjusted data showed a significant association between RBCT and BPD. The odds of BPD among infants having RBCT were 4.01 times higher than the odds of those without transfusion with statistically significant adjusted estimate (OR 5.11, 95% CI, 3.11–8.40). These estimate association between RBCT and BPD for different numbers of transfusions did not differ between groups. Our study provides data on the total volume of transfusions, and the number of transfusions between BPD and non-BPD groups.

The relationship between RBCT and BPD may be explained by several mechanisms. When immature lungs are exposed to a hyperoxic environment, this could lead to a disruption of the alveolar-capillary barrier, overexpression of inflammatory mediators, vascular leakage, and cell death (42). RBCT increases adult hemoglobin in neonates, and shifts the oxygen disassociation curve to the right, increasing lung susceptibility to hyperoxia. Additionally, iron levels and other inflammatory mediators in stored blood products can promote free radical generation, infection, and fibrosis (43). Patel et al. (33) reported that very low BW infants undergoing multiple RBCT had excessive iron stores compared to non-transfused infants, and those who received more transfusions were more prone to developing BPD. This finding supports the hypothesis that free radicals and iron overload play key roles in the pathogenesis of BPD. Evidence also suggests that RBCT can potentially impact the lungs *via* an acute inflammatory response (28). Some neonates that received RBCT required mechanical ventilation, and the ventilator parameters could not be reduced in these patients (28). The hypothesis of transfusion related acute lung injury may be explained by this fact. The production of inflammatory cytokines and immunoactivation of the endothelium observed after the transfusion of RBCT in preterm infants may be a direct causal link between transfusion and major neonatal morbidity (44).

Investigations exploring the association between RBCT and BPD should consider several other factors based on our study. First, the number or the volume of transfusions showed substantial heterogeneity across studies, ranging from one to more than seven. We grouped the studies according to the number of transfusions performed. Although the ORs of BPD did not differ within the groups, heterogeneity was significantly reduced in each subgroup. As hypothesized, a higher number of transfusions may increase the risk of BPD. This might be explained by the small number of studies and variables that were adjusted differently between studies. The second factor that could have influenced the results on the association between RBCT and BPD may be the timing of transfusion. Duan et al. (27) investigated the risk factors for BPD and showed that early but not late anemia increased the risk of BPD. Early exposure to free radicals and iron load during this critical period would influence lung development. However, none of the included studies that described RBCT provided information about the timing of transfusion.

RBCT in neonates could be a lifesaving intervention in certain emergencies, but it is also known to cause adverse events, such as dissemination of infectious disease and inappropriate immune responses (45). Our findings raise the awareness of the risks associated with RBCT. This is the first study to systematically review pooled evidence for an association between BPD and RBCT. We used rigorous methods to search for potential studies, select studies, extract data, and pool the results to identify potential confounders. However, our study has a few limitations. The GA and BW from the included studies varied, and there was limited data to perform subgroup analysis with these parameters. Additionally, the definition of BPD differed among included studies, and misclassification of BPD could lead to possible bias. Primary outcomes in the majority of included studies did not investigate the association between BPD and RBCT, therefore, they may not have adequately controlled for potential confounders such as neonatal co-morbidities. Because more ill neonates often require closer monitoring and have more complications of prematurity and longer hospital stays, more RBCT and higher rates of BPD will be observed in these patients. Furthermore, although we performed a subgroup analysis of crude and adjusted analyses, the adjusted variables were not consistent between studies. There were insufficient data to perform meta-regression to identify potential effectors. The number of studies was lower than needed to interpret the results of subgroup analysis.

## Conclusion

This is the most comprehensive review and analysis of the evidence on the relationship between RBCT and BPD. However, substantial heterogeneity across the body of studies was clearly identified. Many aspects, such as lack of or inadequate confounder adjustments, severity of BPD, and the number of transfusions significantly varied. The association between RBCT and BPD needs more studies to confirmit in the future. This review provides empirical evidence that demonstrating the importance of accounting for potential confounders when interpreting data in this area.

## Data Availability

The original contributions presented in the study are included in the article/**Supplementary Material**, further inquiries can be directed to the corresponding author.

## References

[1] Stoll BJ Hansen NI Bell EF Shankaran LA Walsh MC Neonatal outcomes of extremely preterm infants from the NICHD neonatal research network. Pediatrics. (2010) 126(3):443–56. 10.1542/peds.2009-2959. 20732945 PMC2982806

[2] Keller RL Feng R DeMauro SB Ferkol T Hardie W Rogers EE Bronchopulmonary dysplasia and perinatal characteristics predict 1-year respiratory outcomes in newborns born at extremely low gestational age: a prospective cohort study. J Pediatr. (2017) 187:89–97.e3. 10.1016/j.jpeds.2017.04.026. 28528221 PMC5533632

[3] Hartling L Liang YY Lacaze-Masmonteil T. Chorioamnionitis as a risk factor for bronchopulmonary dysplasia: a systematic review and meta-analysis. Arch Dis Child Fetal Neonatal Ed. (2012) 97(1):F8–F17. 10.1136/adc.2010.210187. 21697236

[4] Bose C Van Marter LJ Laughon M O'Shea TM Allred EN Karna P Fetal growth restriction and chronic lung disease among infants born before the 28th week of gestation. Pediatrics. (2009) 124(3):e450–8. 10.1542/peds.2008-3249 19706590 PMC2891899

[5] Tao Y Han X Guo WL. Predictors of bronchopulmonary dysplasia in 625 neonates with respiratory distress syndrome. J Trop Pediatr. (2022) 68(3):fmac037. 10.1093/tropej/fmac037. 35595255 PMC9122647

[6] Younge N Goldstein RF Bann CM Hintz SR Patel RM Smith PB Survival and neurodevelopmental outcomes among periviable infants. N Engl J Med. (2017) 376(7):617–28. 10.1056/NEJMoa1605566. 28199816 PMC5456289

[7] Stoll BJ Hansen NI Bell EF Walsh MC Carlo WA Shankaran S Trends in care practices, morbidity, and mortality of extremely preterm neonates, 1993–2012. JAMA. (2015) 314(10):1039–51. 10.1001/jama.2015.10244. 26348753 PMC4787615

[8] Doyle LW Carse E Adams AM Ranganathan S Opie G Cheong JLY Ventilation in extremely preterm infants and respiratory function at 8 years. N Engl J Med. (2017) 377(4):329–37. 10.1056/NEJMoa1700827. 28745986

[9] Keir AK Yang J Harrison A Pelausa E Shah PS; Canadian Neonatal Network. Temporal changes in blood product usage in preterm neonates born at less than 30 weeks’ gestation in Canada. Transfusion. (2015) 55(6):1340–6. 10.1111/trf.12998 25652740

[10] Banerjee J Asamoah FK Singhvi D Kwan AWG Morris JK Aladangady N. Haemoglobin level at birth is associated with short term outcomes and mortality in preterm infants. BMC Med. (2015) 13:16. 10.1186/s12916-014-0247-6. 25622597 PMC4307132

[11] Howarth C Banerjee J Aladangady N. Red blood cell transfusion in preterm infants: current evidence and controversies. Neonatology. (2018) 114(1):7–16. 10.1159/000486584. 29550819

[12] Baer VL Lambert DK Henry E Snow GL Christensen RD. Red blood cell transfusion of preterm neonates with a grade 1 intraventricular hemorrhage is associated with extension to a grade 3 or 4 hemorrhage. Transfusion. (2011) 51(9):1933–9. 10.1111/j.1537-2995.2011.03081.x. 21382049

[13] Hengartner T Adams M Pfister RE Snyers D McDougall J Waldvogel S Associations between red blood cell and platelet transfusions and retinopathy of prematurity. Neonatology. (2020) 117(5):1–7. 10.1159/000512020. 33291117 PMC7845415

[14] Marin T Moore J Kosmetatos N Roback JD Weiss P Higgins M Red blood cell transfusion-related necrotizing enterocolitis in very-low-birthweight infants: a near-infrared spectroscopy investigation. Transfusion. (2013) 53(11):2650–8. 10.1111/trf.12158. 23480548 PMC3686850

[15] Lee EY Kim SS Park GY Lee SH. Effect of red blood cell transfusion on short-term outcomes in very low birth weight infants. Clin Exp Pediatr. (2020) 63(2):56–62. 10.3345/kjp.2019.00990. 32024329 PMC7029666

[16] Raffa LH Aljohani W. Evaluation of the effect of blood transfusion on retinopathy of prematurity at a tertiary care center in western Saudi Arabia. Cureus. (2022) 14(4):e24495. 10.7759/cureus.24495. 35651468 PMC9135590

[17] Duan J Kong XY Li QP Hua S Zhang S Feng Z Association between hemoglobin levels in the first 3 days of life and bronchopulmonary dysplasia in preterm infants. Am J Perinatol. (2016) 33(10):998–1002. 10.1055/s-0036-1583189. 27120476

[18] Vecchio AD Henry E D'Amato G Cannuscio A Corriero L Motta M Annamaria cannuscio instituting a program to reduce the erythrocyte transfusion rate was accompanied by reductions in the incidence of bronchopulmonary dysplasia, retinopathy of prematurity and necrotizing enterocolitis. J Matern Fetal Neonatal Med. (2013) 26(Suppl 2):77–9. 10.3109/14767058.2013.830836. 24059559

[19] Demirel N Bas AY Zenciroglu A. Bronchopulmonary dysplasia in very low birth weight infants. Indian J Pediatr. (2009) 76(7):695–8. 10.1007/s12098-009-0110-5. 19381510

[20] Go H Ohto H Nollet KE Sato K Ichikawa H Kume Y Red cell distribution width as a predictor for bronchopulmonary dysplasia in premature infants. Sci Rep. (2021) 11(1):7221. 10.1038/s41598-021-86752-. 33790386 PMC8012706

[21] Stroup DF Berlin JA Morton SC Olkin I Williamson GD Rennie D Meta-analysis of observational studies in epidemiology: a proposal for reporting. Meta-analysis of observational studies in epidemiology (MOOSE) group. Jama. (2000) 283(15):2008–12. 10.1001/jama.283.15.2008. 10789670

[22] Moher D Liberati A Tetzlaff J Altman DG. Preferred reporting items for systematic reviews and meta-analyses: the PRISMA statement. PLoS Med. (2009) 6(7):e1000097. 10.1371/journal.pmed.1000097. 19621072 PMC2707599

[23] Wells GA Shea B O’Connell D Ottawa Hospital Research Institute. Available at: http://www.ohri.ca/programs/clinical_epidemiology/oxford.asp (Accessed 2 May, 2022).

[24] Modesti PA Reboldi G Cappuccio FP Agyemang C Remuzzi G Rapi S Panethnic differences in blood pressure in Europe: a systematic review and meta-analysis. PLoS One. (2016) 11(1) :e0147601. 10.1371/journal.pone.0147601 26808317 PMC4725677

[25] Hozo SP Djulbegovic B Hozo I. Estimating the mean and variance from the median, range, and the size of a sample. BMC Med Res Methodol. (2005) 5:13. 10.1186/1471-2288-5-13. 15840177 PMC1097734

[26] Liao ZX Zhao X Rao HP Kang Y. Analysis of correlative risk factors for blood transfusion therapy for extremely low birth weight infants and extreme preterm infants. Am J Transl Res. (2021) 13(7):8179–85. eCollection 2021. 10.1038/srep22717 34377303 PMC8340198

[27] Duan J Kong XY Li QP Hua S Zhang S Zhang X. Association between anemia and bronchopulmonary dysplasia in preterm infants. Sci Rep. (2016) 6:22717. 10.1038/srep22717. 26936610 PMC4776173

[28] Zhang ZQ Huang XM Lu H. Association between red blood cell transfusion and bronchopulmonary dysplasia in preterm infants. Sci Rep. (2014) 4:4340. 10.1038/srep04340. 24614152 PMC3949297

[29] Cai HW Jiang L Liu YS Shen T Yang Z Wang S Development and verification of a risk prediction model for bronchopulmonary dysplasia in very low birth weight infants. Transl Pediatr. (2021) 10(10):2533–43. 10.21037/tp-21-445. 34765477 PMC8578781

[30] Sharma A Xin YM Chen XG Sood BG. Early prediction of moderate to severe bronchopulmonary dysplasia in extremely premature infants. Pediatr Neonatol. (2020) 61(3):290–9. 10.1016/j.pedneo.2019.12.001. 32217025

[31] Ghirardello S Dusi E Cortinovis I Villa S Fumagalli M Agosti M Effects of red blood cell transfusions on the risk of developing complications or death: an observational study of a cohort of very low birth weight infants. Am J Perinatol. (2017) 34(1):88–95. 10.1055/s-0036-158430. 27249797

[32] Valieva OA Strandjord TP Mayock DE Juul SE. Effects of transfusions in extremely low birth weight infants: a retrospective study. J Pediatr. (2009) 155(3):331–37.e1. 10.1016/j.jpeds.2009.02.026. 19732577 PMC3038786

[33] Patel RM Knezevic A Yang J Shenvi N Hinkes M Roback JD Enteral iron supplementation, red blood cell transfusion, and risk of bronchopulmonary dysplasia in very-low-birth-weight infants. Transfusion. (2019) 59(5):1675–82. 10.1111/trf.15216. 30801736 PMC6499698

[34] Korhonen P Tammela O Koivisto AM Laippala P Ikonen S. Frequency and risk factors in bronchopulmonary dysplasia in a cohort of very low birth weight infants. Early Hum Dev. (1999) 54(3):245–58. 10.1016/s0378-3782(98)00101-7. 10321791

[35] Jassem-Bobowicz JM Klasa-Mazurkiewicz D Żawrocki A Stefańska K Domżalska-Popadiuk I Kwiatkowski S Prediction model for bronchopulmonary dysplasia in preterm newborns. Children (Basel). (2021) 8(10):886. 10.3390/children8100886. 34682151 PMC8534367

[36] Soliman N Chaput K Alshaikh B Yusuf K. Preeclampsia and the risk of bronchopulmonary dysplasia in preterm infants less than 32 Weeks’ gestation. Am J Perinatol. (2017) 34(6):585–92. 10.1055/s-0036-1594017. 27919118

[37] Zhang HS Fang JP Su HB Risk factors for bronchopulmonary dysplasia in neonates born at ≤ 1500 g (1999-2009). Pediatr Int. (2011) 53(6):915–20. 21605281 10.1111/j.1442-200X.2011.03399.x

[38] Jeon GW Sin JB. Risk factors of transfusion in anemia of very low birth weight infants. Yonsei Med J. (2011) 53(6):915–20. 10.1111/j.1442-200X.2011.03399.x. PMC3575997 23364969

[39] Gao SQ Xiao TT Ju R Ma R Zhang X Dong W. The application value of lung ultrasound findings in preterm infants with bronchopulmonary dysplasia. Transl Pediatr. (2020) 9(2):93–100. 10.21037/tp.2020.03.14. 32477908 PMC7237972

[40] Park SH Kim HM. The iron Status of very low birth weight infants receiving multiple erythrocyte transfusions during hospitalization in the neonatal intensive care unit. Pediatr Gastroenterol Hepatol Nutr. (2015) 18(2):100–7. 10.5223/pghn.2015.18.2.100. 26157695 PMC4493242

[41] Lardón-Fernández M Uberos J Molina-Oya M Narbona-López E. Epidemiological factors involved in the development of bronchopulmonary dysplasia in very low birth-weight preterm infants. Minerva Pediatr. (2017) 69(1):42–9. 10.23736/S0026-4946.16.04215-8. 25715027

[42] Harijith AK Bhandari V. Hyperoxia in the pathogenesis of bronchopulmonary dysplasia. In: Bhandari V, editors. Bronchopulmonary dysplasia. Respiratory medicine. Cham: Humana Press (2016). p. 3–26. 10.1007/978-3-319-28486-6_1

[43] Collard KJ. Is there a causal relationship between the receipt of blood transfusions and the development of chronic lung disease of prematurity? Med Hypotheses. (2006) 66(2):355–64. 10.1016/j.mehy.2005.04.046. 16236459

[44] Keir AK McPhee AJ Andersen CC Stark MJ. Plasma cytokines and markers of endothelial activation increase after packed red blood cell transfusion in the preterm infant. Pediatr Res. (2013) 73(1):75–9. 10.1038/pr.2012.14. 23095979

[45] Howarth C Banerjee J Aladangady N. Red blood cell transfusion in preterm infants: current evidence and controversies. Neonatology. Neonatology. (2018) 114(1):7–16. 10.1159/0004865. 29550819

