# Supplementary material for: Association between red blood cell transfusion and bronchopulmonary dysplasia: a systematic review and meta-analysis
Source: Front Pediatr. 2023 May 31;11:1095889. doi: 10.3389/fped.2023.1095889 (PMC10266411; doi:10.3389/fped.2023.1095889)
Supplement: Supplementary file 1 [file Datasheet1.docx]

**TITLE** Red blood cell transfusion as a risk factor for bronchopulmonary dysplasia: A Systematic Review and Meta-Analysis

**Contents**

Diagram 1. Flow chart of the literature search and selection process------------page 2

Search strategy--------------------------------------------------------------------------page 3

sTable 1-2. Raw data of included studies reporting the number of red blood cell transfusions

and total volume of red blood cell transfusions -----------------------------------page 4

Quality score of included studies--------------------------------------------------- page 5-6

sFigure 2. Forest plot for the number of RBCT and total volume of transfusions between

BPD patient and control-------------------------------------------------------------- page 7

sTable 3.Variable adjusted in the studies included---------------------------------- page 8

**Diagram 1. Flow chart of the literature search and selection process**

## Identification

## Eligibility

## Included

## Screening

Additional records identified through other sources
(n = 14)

Records identified through database searching
PubMed (n = 205) Web of Science (n = 212) EMBASE (580)

Records from study search
(n =1011)

Records excluded for duplicated studies
(n =235)

Records screened
(n =776)

Records excluded for clearly irrelevant, review, case reports
(n = 736)

Full-text articles excluded, with reasons (n = 19)

No BPD data (12)

No transfusion data (5)

No comparion group (2)

Overlapping populations (2)

Full-text articles assessed for eligibility
(n = 40)

Studies included in quantitative synthesis (meta-analysis)
(n = 21)

**Electronic database search strategy**

**Pubmed**

**Limit to English**

(("Blood Transfusion"[Mesh]) OR (“blood transfusion”[Title/Abstract]) OR (“blood transfusions”[Title/Abstract]) OR (“blood component transfusion”[Title/Abstract]) OR (“blood component transfusions”[Title/Abstract]) OR (“Erythrocyte Transfusion”[Title/Abstract]) OR (“Erythrocyte Transfusions”[Title/Abstract]) OR (“red blood cell transfusion”[Title/Abstract]) OR (“red blood cell transfusions”[Title/Abstract]) OR (“red cell transfusion”[Title/Abstract]) OR (“red cell transfusions”[Title/Abstract]) AND ("bronchopulmonary dysplasia"[MeSH Terms] OR" bronchopulmonary dysplasia "[Title/Abstract] OR " BPD"[Title/Abstract] OR "chronic lung disease "[Title/Abstract] OR "CLD "[Title/Abstract])

**Web of Science**

**Limit to English**

((“blood transfusion” OR “red blood cell transfusions” OR “Erythrocyte Transfusions”) AND (“bronchopulmonary dysplasia” OR “BPD” OR “chronic lung disease” OR“CLD”) )

**Ovid EMBASE**

**Limit to Human and English**

((“bronchopulmonary dysplasia”, OR “chronic lung disease” OR “BPD” OR “CLD”) AND (“blood cell transfusion” OR “Erythrocyte Transfusions” OR “red blood cell transfusions”))

| **sTable 1. Raw data of included studies reporting the number of red blood cell transfusions** | | | | | | |
| --- | --- | --- | --- | --- | --- | --- |
| **Study** | **BPD** | | | **Non-BPD** | | |
| number of red blood cell transfusions | Mean | SD | Number | Mean | SD | Number |
| Duan [25] | 5.4 | 2.7 | 71 | 2.1 | 1.3 | 172 |
| Raffa[14] | 3.6 | 2.4 | 5 | 3.04 | 2.6 | 106 |
| Go[18] | 2.4 | 4.5 | 85 | 2.4 | 4.5 | 91 |
| Zhang[36] | 4.4 | 2.9 | 56 | 1.8 | 1 | 60 |
| Park[39] | 10.6 | 4.9 | 10 | 2.2 | 1.9 | 36 |

| **sTable 2. Raw data of included studies reporting the** **total volume of red blood cell transfusions** | | | | | | |
| --- | --- | --- | --- | --- | --- | --- |
| **Study** | **BPD** | | | **Non-BPD** | | |
| total volume of red blood cell transfusions | Mean | SD | Number | Mean | SD | Number |
| Patel[32] | 62 | 60.4 | 240 | 7.7 | 16.3 | 358 |
| Raffa[14] | 52.6 | 15.9 | 5 | 54.9 | 54.2 | 106 |
| Park[39] | 191.1 | 93.5 | 10 | 50.2 | 43.2 | 36 |

**The Detailed Assessment Process of Every Included Article (NOS)**

|  |  |  |  |  |  |  |  |  |  |  |
| --- | --- | --- | --- | --- | --- | --- | --- | --- | --- | --- |
| Cohort study | | Selection 1) | Selection 2) | Selection 3) | Selection 4) | Comparability 1) | Outcome 1) | Outcome 2) | Outcome 3) | Score |
| Zhang[26] | | a) | a) | a) | a) | a) | b) | a) | a) | 8 |
| Ghirardello [30] | | a) | a) | a) | a) | - | b) | a) | a) | 7 |
| Patel[32] | | a) | a) | a) | a) | a) | b) | a) | d) | 7 |
| Raffa[14] | | b) | a) | a) | a) | - | b) | a) | d) | 6 |
| Korhonen[33] | | a) | a) | a) | a) | - | b) | b) | b) | 5 |
| Tao[5] | | c) | a) | a) | a) | a) | b) | a) | d) | 6 |
| Soliman[35] | | b) | a) | a) | a) | - | b) | a) | d) | 6 |
| Go[18] | | a) | a) | a) | a) | a) | b) | a) | d) | 7 |
| LARDÓN-FERNÁNDEZ[39] | | a) | a) | a) | a) | a) | b) | a) | d) | 7 |

|  |  |  |  |  |  |  |  |  |  |  |
| --- | --- | --- | --- | --- | --- | --- | --- | --- | --- | --- |
| Case-control study | | Selection 1) | Selection 2) | Selection 3) | Selection 4) | Comparability 1) | Exposure 1) | Exposure 2) | Exposure 3) | Score |
| Liao[24] | | a) | a) | a) | a) | - | d) | a) | b) | 5 |
| Demirel[17] | | a) | a) | a) | a) | a) | e) | a) | b) | 6 |

| Cross-sectional study | Selection  A. All subjects or random sampling (3) ;B. Non-random sampling (2) ; C. Selected group of users (1)  D. No description of sampling strategy (0) | Selection (sample size)  A. Justified and satisfactory (1)  B. Not justified (0) | Detection (outcome measurement)  A. Validated measurement tool (2); B. Tool described but non-validated (1) ;C. Tool not described (0) | Confounding  A. Adjusted for confounders (1)  B. No adjustment for confounders (0) | Outcome  A. Independent blind assessment (1)  B. Record linkage (0)  C. Self-report (0)  D. No description (0) | Score |
| --- | --- | --- | --- | --- | --- | --- |
| Duan [25] | 1 | 1 | 2 | 1 | 0 | 5 |
| Cai [28] | 1 | 1 | 2 | 1 | 0 | 5 |
| Sharma [29] | 1 | 0 | 2 | 1 | 0 | 4 |
| Lee [13] | 1 | 0 | 2 | 1 | 0 | 4 |
| Valieva[31] | 1 | 0 | 2 | 1 | 0 | 4 |
| Jassem-Bobowicz[34] | 1 | 0 | 2 | 1 | 0 | 4 |
| Zhang[35] | 1 | 0 | 2 | 1 | 0 | 4 |
| Jeon[36] | 1 | 0 | 2 | 1 | 0 | 4 |
| Gao[37] | 1 | 0 | 2 | 1 | 0 | 4 |
| Park[38] | 1 | 0 | 2 | 1 | 0 | 4 |

**sFigure 2. Forest plot for the number of RBCT and total volume of transfusions between**

**BPD patient and control. A number of RBCT. B total volume of transfusions.**

**
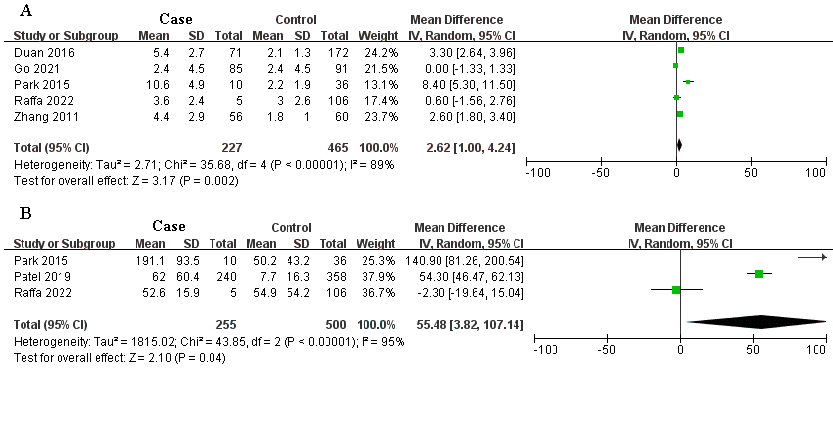
**

| sTable 3.Variable adjusted in the studies included | |
| --- | --- |
| Ref | Adjustments |
| Korhonen,1988 | Preecclampsia, BW, Duration of ventilator therapy, surfactant, PDA, hyperoxia, |
| LARDÓN-FERNÁNDEZ 2017 | GA,BW, Chorioamnionitis, Duration of mechanical ventilation and non-invasive mechanical ventilation; time of parenteral nutrition and enteral feeding; use of erythropoietin,hyaline membrane disease, Pneumothorax, Prenatal and postnatal corticosteroids |
| Lee,2020 | gestational age, birth weight, Apgar score at 1 minute, RDS,Sepsis,NEC,IVH,ROP, Days to reach full feeding |
| Sharma 2019 | BW, Sex, doses of surfactant, maximal FiO2>40% on day 1, cumulative MV for  7 days in first 21 days after birth |
| Solima 2016 | Preclampsia, Sex, surfactant,IUGR,Sepsis |
| Tao, 2022 | BW,GA<32w,total duration of oxygen therapy, neonatal asphyxia, PDA, surfactant |
| Zhang,2014 | GA, BW, transfusion PRBC, sepsis, aminophylline use, NEC, no surfactant use, and mechanical ventilation > 1 week |
| Zhang 2011 | GA<30 weeks, Maternal chorioamnionitis, ventilation-associated pneumonia. |
